# Fluid overload at start of continuous renal replacement therapy is associated with poorer clinical condition and outcome: a prospective observational study on the combined use of bioimpedance vector analysis and serum N-terminal pro-B-type natriuretic peptide measurement

**DOI:** 10.1186/s13054-015-0871-3

**Published:** 2015-04-02

**Authors:** Haiyan Chen, Buyun Wu, Dehua Gong, Zhihong Liu

**Affiliations:** National Clinical Research Center of Kidney Disease, Jinling Hospital, Nanjing University School of Medicine, Zhongshan East Road 305#, Nanjing, 210016 P. R. China

## Abstract

**Introduction:**

It is unclear whether the fluid status, as determined by bioimpedance vector analysis (BIVA) combined with serum N-terminal pro-B-type natriuretic peptides (NT-pro-BNP) measurement, is associated with treatment outcome among patients receiving continuous renal replacement therapy (CRRT). Our objective was to answer this question.

**Methods:**

Patients who were in the intensive care units of a university teaching hospital and who required CRRT were screened for enrollment. For the enrolled patients, BIVA and serum NT-pro BNP measurement were performed just before the start of CRRT and 3 days afterward. According to the BIVA and NT-pro BNP measurement results, the patients were divided into four groups according to fluid status type: type 1, both normal; type 2, normal BIVA results and abnormal NT-pro BNP levels; type 3, abnormal BIVA results and normal NT-pro BNP levels; and type 4, both abnormal. The associations between fluid status and outcome were analyzed.

**Results:**

Eighty-nine patients were enrolled, 58 were males, and the mean age was 49.0 ± 17.2 years. The mean score of Acute Physiology and Chronic Health Evaluation II (APACHE II) was 18.8 ± 8.6. The fluid status before CRRT start was as follows: type 1, 21.3% (19 out of 89); type 2, 16.9% (15 out of 89); type 3, 11.2% (10 out of 89); and type 4, 50.6% (45 out of 89). There were significant differences between fluid status types before starting CRRT on baseline values for APACHE II scores, serum creatinine, hemoglobin, platelet count, urine volume, and incidences of oliguria and acute kidney injury (*P* <0.05). There were significant differences between patients with different fluid status before CRRT start on hospital mortality—type 1, 26.3% (5 out of 19); type 2, 33.3% (5 out of 15); type 3, 40% (4 out of 10); and type 4, 64.4% (29 out of 45) (*P* = 0.019)—as well as renal function recovery rates: type 1, 57.1% (4 out of 7); type 2, 67.7% (6 out of 9); type 3, 50% (3 out of 6); and type 4, 23.7% (9 out of 38) (*P* = 0.051).

**Conclusions:**

Fluid status abnormalities were common among patients receiving CRRT. Different types of fluid status distinguished by BIVA combined with serum NT-pro BNP measurements corresponded to different clinical conditions and treatment outcomes, which implies a value of this method for evaluation of fluid status among patients receiving CRRT.

**Electronic supplementary material:**

The online version of this article (doi:10.1186/s13054-015-0871-3) contains supplementary material, which is available to authorized users.

## Introduction

Fluid overload is a risk factor for mortality among critically ill patients. As reported in an observational study using data generated by the RENAL (Randomized Evaluation of Normal versus Augmented Level) trial, a negative mean daily fluid balance was consistently associated with improved clinical outcomes [1]. For patients receiving continuous renal replacement therapy (CRRT) due to the loss of renal capacity, fluid balance is largely dependent on decisions of the attending physician which are based on an accurate fluid status assessment. However, there is no consensus on an effective method to evaluate fluid status.

The most commonly used methods to calculate daily fluid balance, such as monitoring changes in body weight, are obviously imprecise with pronounced diversity between different methods [2]. Bioimpedance vector analysis (BIVA) is a recently proposed method for fluid status assessment because of the advantages of non-invasiveness, convenience, low cost, real-time measurements, and good reproducibility [3,4]. Theoretically, BIVA reflects tissue hydration (that is, static fluid load), which is not always in accordance with blood volume or cardiac function. Serum N-terminal pro-B-type natriuretic peptide (NT-pro BNP), which is produced by cardiomyocytes under stretching stress, reflects cardiac reaction to volume load and acts as a biomarker for diagnosis of heart failure [5]. However, serum NT-pro BNP does not reflect tissue hydration status. Therefore, the differentiation of ‘wet BNP’ (induced by acute pressure or volume overload) from ‘dry BNP’ (baseline, euvolemic) has been suggested to improve assessment of fluid status in clinical practice [6]. Apparently, either BIVA or NT-pro BNP only partially reflects fluid status; thus, the combined use of these parameters may offer more precise information about fluid status. However, there are no data on the combined use of BIVA and NT-pro BNP to assess fluid status in patients receiving CRRT.

In the present study, we assessed fluid status among patients receiving CRRT by using BIVA combined with serum NT-pro BNP and identified correlations between outcome and fluid status, as determined by the combination of these two parameters.

## Methods

### Patients

From April 2013 to March 2014, all patients requiring CRRT in Jinling Hospital (Nanjing University School of Medicine, Nanjing, China) were screened for enrollment. The inclusion criteria were age of more than 18 years, admission to the intensive care unit, and the requirement of CRRT, and the exclusion criteria were the presence of malignant disease, pregnancy, history of end-stage renal disease, and burn injury. The study protocol was previously approved by the Ethics Committee of Jinling Hospital, and informed consent was obtained from each patient or next of kin before participation. The treatment protocol, including CRRT and prescribed medications to maintain volume balance, remained unchanged after enrollment.

### Continuous renal replacement therapy protocol

A central venous catheter was used for blood access at a blood flow rate of 160 to 200 mL/minute. Pre-dilution continuous hemofiltration (CVVH) was performed by using a polysulphone filter (AV600S; Fresenius SE & Co. KGaA, Bad Homburg, Germany), with a replacement fluid infusion rate of 2,000 to 3,000 mL/hour. Regional citrate anticoagulation combined with low-dose heparin was used to maintain activated clotting time within the extracorporeal circuit in a desired range (200 to 250 seconds). Daily net ultrafiltration volume was determined by the attending physician.

### Data collection

BIVA evaluation and serum NT-pro BNP measurement were performed for all participants just before the start of CRRT and 3 days later. The clinical status, incidence of acute kidney injury (AKI), hospital mortality, and renal function recovery were recorded. Right-sided whole-body impedance, including resistance and reactance, was measured by using a Bodystat QuadScan 4000 device (Bodystat Ltd., Isle of Man, UK) at a frequency of 50 kHz. In accordance with the instructions of the manufacturer, a tetrapolar wrist-to-ankle method was used, in which a pair of electrodes was placed approximately 5 mm apart on the dorsal surface of the right wrist and ipsilateral ankle, with the patient in the supine position. The hydration status was determined by plotting the point vector on the reference bivariate tolerance ellipses (RXc point graph). Vector displacements parallel to the major axis of tolerance ellipses indicate progressive changes in tissue hydration, in which short vectors out of the lower pole (falling out of the 75% tolerance ellipse) indicate overhydration [7]. Serum NT-Pro BNP was detected in our hospital laboratory by using electrochemical luminescence immunoassay analysis (Cobas assay; Roche Diagnostics, Mannheim, Germany), with a level of more than 106.2 pmol/L (the normal range provided by the hospital lab) considered abnormal. AKI was diagnosed according to the KDIGO (Kidney Disease: Improving Global Outcomes) criteria [8], and renal recovery was defined as a return of creatinine to pre-morbid renal function levels (serum creatinine (sCr) <1.5 × premorbid sCr) within 3 months.

### Fluid status assessment

According to the results of BIVA and serum NT-pro BNP measurements, fluid status was divided into four types: type 1, normal status, neither overhydration nor elevation of NT-pro BNP; type 2, no overhydration with abnormal NT-pro BNP levels; type 3, overhydration with normal NT-pro BNP levels; and type 4, overhydration with abnormal NT-pro BNP levels.

### Statistical analysis

Continuous variables with a normal distribution are presented as the mean (± standard deviation) and compared between groups by using the Student’s *t* test. Continuous variables with a skewed distribution are presented as the median (range) and compared between groups by using the rank-sum test. Categorical variables are expressed as number (percentage) and compared between groups by using the chi-squared test. Survival curves were generated by using the Kaplan-Meier method and compared by using the log-rank test. Univariate and multivariate adjusted logistic regression analyses were performed to determine whether NT-ProBNP, BIVA, or both was an independent risk factor for mortality. Data were analyzed by using SAS version 9.2 statistical software (SAS Institute Inc., Cary, NC, USA). A two-sided *P* value of less than 0.05 was considered statistically significant.

## Results

In total, 89 patients, including 58 males, with a mean age of 49.0 ± 17.2 years were enrolled in this study. The primary diseases were as follows (Table 1): intestinal fistula and associated intra-abdominal infection (n = 27, 30.3%), severe acute pancreatitis (n = 28, 31.5%), trauma (n = 8, 9.0%), acute liver failure (n = 4, 4.5%), pulmonary infection (n = 9, 10.1%), and other diseases (n = 13, 14.6%). Among them, the incidences of multiple organ dysfunction syndrome, sepsis, and AKI were 76.4% (n = 68), 21.3% (n = 19), and 67.4% (n = 60), respectively. The mean score of the Acute Physiology and Chronic Health Evaluation II (APACHE II) was 18.8 ± 8.6. The mean CRRT duration was 8.0 ± 8.2 days. Among the 60 patients with AKI, 22 (36.7%) achieved recovery of renal function. Forty-three (48.3%) patients died before discharge. Seventy patients completed second volume status evaluations, and the others dropped out within 3 days after the start of CRRT, which included 15 who died and four who were discharged.

**Table 1:** **Clinical conditions between different types of fluid status before continuous renal replacement therapy**

**Fluid status**	**Total**	**Type 1**	**Type 2**	**Type 3**	**Type 4**	***P*** **value**
Number (%)	89 (100)	19 (21.3)	15 (16.9)	10 (11.2)	45 (50.6)	
Male/female	58/31	13/6	9/6	9/1	27/18	0.319
Age, years	49.0 ± 17.2	45.5 ± 15.1	44.7 ± 16.6	43.3 ± 17.9	53.1 ± 17.7	0.148
Height, cm	167.9 ± 10.4	168.5 ± 7.0	166.2 ± 8.8	176.9 ± 6.2	166.4 ± 12.0	0.015
Weight, kg	70.4 ± 15.8	69.0 ± 23.1	63.2 ± 10.0	78.5 ± 14.5	72.0 ± 12.7	0.023
Primary reason for hospitalization, n (%)						
Pancreatic	28 (31.4)	11 (57.9)	2 (13.3)	4 (40.0)	11 (24.4)	0.021
Gastrointestinal	27 (30.3)	5 (26.3)	3 (20.0)	4 (40.0)	15 (33.3)	0.678
Liver	4 (4.5)	2 (10.5)	1 (6.7)	0	1 (2.2)	0.431
Pulmonary	9 (10.1)	0	3 (20.0)	1 (10.0)	5 (11.1)	0.289
Trauma	8 (9.0)	0	1 (6.7)	1 (10.0)	6 (13.3)	0.393
Other	13 (9.0)	1 (5.3)	5 (33.3)	0	7 (15.5)	0.066
Acute kidney injury, n (%)	60 (67.4)	7 (36.8)	9 (60.0)	6 (60.0)	38 (84.4)	0.002
Acute heart failure, n (%)	24 (27.0)	0	4 (26.7)	1 (10.0)	19 (42.2)	0.003
Severe sepsis, n (%)	63 (70.8)	10 (52.6)	8 (53.3)	9 (90.0)	36 (80.0)	0.032
Mechanical ventilation, n (%)	54 (60.7)	6 (31.6)	7 (46.7)	6 (60.0)	36 (80.0)	0.002
Comorbidities, n (%)						
Diabetes mellitus	11 (12.3)	2 (10.5)	3 (20.0)	0	6 (13.3)	0.514
Chronic heart failure	4 (4.5)	0	2 (13.3)	0	2 (4.4)	0.256
Chronic renal failure	7 (7.9)	0	6 (40.0)	0	1 (2.2)	<0.001
Urea, mmol/L	17.9 ± 12.7	13.6 ± 13.9	18.3 ± 12.6	16.3 ± 14.3	20.1 ± 11.8	0.301
Creatinine, μmol/L	287.5 ± 290.0	119.6 ± 138.3	496.7 ± 457.3	176.5 ± 191.0	313.2 ± 236.1	0.001
Oliguria, n (%)	45 (50.6%)	3 (15.8%)	11 (73.3%)	5 (50%)	26 (57.8%)	0.004
Urine output, mL/day	803 ± 796	1,309 ± 652	429 ± 388	916 ± 755	732 ± 878	0.008
Hemoglobin, g/L	103.5 ± 32.4	121.1 ± 34.2	109.9 ± 40.7	96.8 ± 16.0	95.5 ± 28.6	0.022
Platelet, ×10^9^/L	136.0 ± 88.7	186.5 ± 70.2	165.7 ± 114.7	106.7 ± 75.7	111.2 ± 78.3	0.005
CRP, mg/L	133.4 ± 78.0	114.3 ± 70.4	105.4 ± 77.3	165.8 ± 73.2	141.7 ± 80.0	0.177
Albumin, g/L	30.3 ± 4.9	29.2 ± 5.1	32 ± 6.8	29.7 ± 3.5	30.3 ± 4.4	0.419
Bilirubin, mmol/L^a^	27.6 (14.4-37.8)	23.3 (17.3-51.4)	10.0 (6.4-64.8)	45.5 (16.9-100.3)	27.6 (16.3-85.4)	0.304
Lactate, mmol/L^a^	2.1 (1.3-3.8)	2.1 (1.5-3.4)	1.9 (1.3-3.8)	1.8 (1.3-3.5)	2.4 (1.3-3.9)	0.952
NT-ProBNP, pmol/L^a^	274 (62-1,491)	15.4 (8.5-54)	591 (203-1,949)	61 (51-65)	817 (294-2,549)	<0.001
APACHE II score	18.8 ± 8.6	13.6 ± 6.7	17.1 ± 7.5	15.5 ± 8.4	22.2 ± 8.3	<0.001
SOFA score	11.7 ± 6.0	6.6 ± 3.0	10.6 ± 5.1	10.7 ± 6.4	14.6 ± 5.6	<0.001
SAPS II	41.7 ± 18.5	19.7 ± 12.7	42.5 ± 18.3	34.8 ± 22.2	48.4 ± 17.0	<0.001
Fluid balance at day 1, mL^a^	1,134 (169-2,301)	966 (210-1,702)	1,734 (602-3,092)	1,514 (670-2,157)	1,118 (−334-2,499)	0.557
Cumulative fluid balance over 3 days, mL^a^	2,729 (676-4,883)	2,729 (676-4,883)	3,843 (2,515-4,824)	2,517 (514-5,571)	2,611(−993-5,282)	0.7935

^a^Median (range). Fluid status: type 1, no overhydration and B-type natriuretic peptide (BNP) normal; type 2, no overhydration but BNP abnormal; type 3, overhydration but BNP normal; type 4, overhydration and BNP abnormal. APACHE II, Acute Physiology and Chronic Health Evaluation II; CRP, C-reactive protein; NT-pro BNP, N-terminal pro-B-type natriuretic peptide; SAPS II, Simplified Acute Physiology Score II; SOFA, Sequential Organ Failure Assessment.

### Correlations between pre-continuous renal replacement therapy fluid status and clinical conditions and outcomes

**Figure 1:**
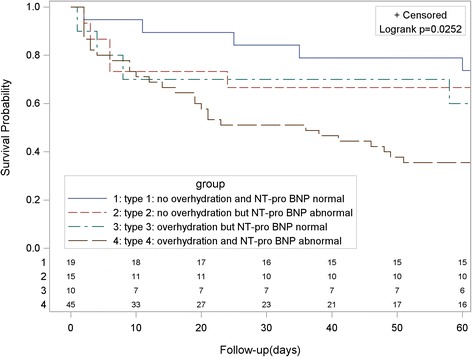
**Kaplan-Meier survival curves for patients with different fluid status at start of continuous renal replacement therapy.** The type of fluid status was determined by combination of bioimpedance vector analysis and serum N-terminal pro-B-type natriuretic peptide (NT-pro-BNP).

The fluid status prior to the start of CRRT was as follows: type 1, 19 cases (21.3%); type 2, 15 cases (16.9%); type 3, 10 cases (11.2%); and type 4, 45 cases (50.6%). As shown in Table 1, the clinical conditions of patients revealed significant differences between types of fluid status according to APACHE II scores, serum creatinine levels, hemoglobin concentrations, platelet counts, urine volume, and incidences of oliguria and AKI (*P* <0.05). Although there were no differences between fluid status types on mortality on day 3, there were significant differences in hospital mortality as well as renal function recovery (Table 2). Kaplan-Meier survival curve analysis revealed that the survival rate of patients classified as type 4 fluid status was lower than in the other groups (Figure 1, *P* = 0.0252).

**Table 2:** **Clinical outcomes between different types of fluid status before continuous renal replacement therapy**

**Fluid status**	**Type 1**	**Type 2**	**Type 3**	**Type 4**	**Total**	***P*** **value**
Number	19	15	10	45	89	
Death within 3 days, n (%)	1 (5.3)	2 (13.3)	2 (20)	10 (22.2)	15 (16.9)	0.399
Death before discharge, n (%)	5 (26.3)	5 (33.3)	4 (40)	29 (64.4)	43 (48.3)	0.019
CRRT duration, day	5.1 ± 4.1	5.2 ± 3.0	8.5 ± 13.0	10.0 ± 8.7	8.0 ± 8.2	0.091
Recovery of renal function, n (%)	4 (57.1)	6 (67.7)	3 (50)	9 (23.7)	22 (36.7)	0.047

Fluid status: type 1, no overhydration and B-type natriuretic peptide (BNP) normal; type 2, no overhydration but BNP abnormal; type 3, overhydration but BNP normal; type 4, overhydration and BNP abnormal. CRRT, continuous renal replacement therapy.

### Changes in fluid status after start of continuous renal replacement therapy

A second fluid status evaluation was performed for 70 patients 3 days after beginning CRRT. Patient mortality according to fluid status on day 3 was as follows: type 1, 25% (4 out of 16), type 2, 25% (3 out of 12), type 3, 40% (4 out of 10), and type 4, 60% (18 out of 30) (Table 3, *P* = 0.051). Similar to the results of primary fluid status, the highest mortality was among patients with type 4 fluid status on day 3. For patients with type 4 fluid status before CRRT, those with continued type 4 fluid status on day 3 had high mortality (14 out of 20), whereas those with improvement in one parameter of fluid status on day 3 had the lowest mortality (1 out of 5 and1 out of 4) and those with improvements in both two parameters of fluid status (change to type 1 fluid status) had the highest mortality (3 out of 4).

**Table 3:** **Changes of fluid status and cumulative fluid balance over 3 days and outcomes (n = 70)**

**Fluid status before CRRT**		**Fluid status 3 days later**				**Total death, n (%)**
		**Type 1**	**Type 2**	**Type 3**	**Type 4**	
Type 1	Cumulative fluid balance, mL	1,664 ± 1,971	3,832 ± 4,291	2,626 ± 3,732	870	
	Death, n (%)	1/10 (10%)	1/3 (33.3%)	2/3 (66.6%)	0/1 (0%)	4/17 (23.5%)
Type 2	Cumulative fluid balance, mL	1,566	2,365 ± 2,140	3,935 ± 2,130	5,974 ± 3,144	
	Death, n (%)	0/1 (0%)	1/4 (25%)	0/2 (0%)	2/4 (50%)	3/11 (27.3%)
Type 3	Cumulative fluid balance, mL	571	-	1,601 ± 1,147	3,242 ± 3,296	
	Death, n (%)	0/1 (0%)	0	1/3 (33.3%)	2/5 (40%)	3/9 (33.3%)
Type 4	Cumulative fluid balance, mL	−1,380 ± 3,308	−1,159 ± 3,394	2,461 ± 2,844	2,613 ± 3,962	
	Death, n (%)	3/4 (75%)	1/5 (20%)	1/4 (25%)	14/20 (70%)	19/33 (57.6%)
Total death, n (%)		4/16 (25%)	3/12 (25%)	4/12 (33.3%)	18/30 (60%)	29/70 (41.4%)

Fluid status: type 1, no overhydration and B-type natriuretic peptide (BNP) normal; type 2, no overhydration but BNP abnormal; type 3, overhydration but BNP normal; type 4, overhydration and BNP abnormal. CRRT, continuous renal replacement therapy.

### Relationship between mortality and N-terminal pro-B-type natriuretic peptide or bioimpedance vector analysis or both

Univariate logistic regression analyses indicated that age, APACHE II score, AKI, acute heart failure, severe sepsis, mechanical ventilation, overhydration on day 1, high NT-ProBNP on day 1, and overhydration plus high NT-ProBNP on day 1 were potential risk factors for mortality. However, in the multivariate adjusted analyses, only APACHE II score was an independent risk factor for mortality (Table 4). Furthermore, multivariate adjusted analyses using NT-Pro BNP and BIVA on day 3 revealed that only APACHE II score was an independent risk factor for mortality (data not shown).

**Table 4:** **Results of logistic regression analysis of risk factors for death (n = 89)**

**Variables**	**Univariate regression**		**Adjusted multivariate regression**	
	**Odds ratio**	***P*** **value**	**Odds ratio**	***P*** **value**
Age	1.04 (1.01-1.07)	0.006	1.02 (0.98-1.05)	0.374
Gender	0.82 (0.34-1.96)	0.649	0.72 (0.21-2.45)	0.597
APACHE II score	1.20 (1.11-1.29)	<0.001	1.18 (1.08-1.29)	<0.001
Acute kidney injury	2.90 (1.14-7.42)	0.026	0.87 (0.19-4.05)	0.863
Acute heart failure	2.82 (1.06-7.51)	0.039	1.84 (0.44-7.81)	0.405
Severe sepsis	3.62 (1.33-9.84)	0.012	3.83 (0.74-19.74)	0.109
Mechanical ventilation	4.50 (1.76-11.47)	0.002	3.05 (0.86-10.88)	0.086
Chronic renal failure	0.79 (0.17-3.74)	0.764	1.04 (0.06-19.44)	0.981
Chronic heart failure	3.38 (0.34-33.76)	0.300	4.66 (0.15-143.72)	0.379
Overhydration at day 1	3.60 (1.44-8.98)	0.006	-	-
High NT-ProBNP at day 1	2.54 (1.02-6.35)	0.046	-	-
Overhydration + high NT-ProBNP at day 1	3.88 (1.61-9.36)	0.002	1.02 (0.30-3.38)	0.997

Variables in the multivariate regression included age, gender, Acute Physiology and Chronic Health Evaluation II (APACHE II) score, acute kidney injury, acute heart failure, severe sepsis, mechanical ventilation, chronic renal failure, chronic heart failure, and presence of overhydration plus high N-terminal pro-B-type natriuretic peptide (NT-ProBNP) level.

## Discussion

In this prospective observational study, fluid status was evaluated by using the combination of BIVA and NT-Pro BNP measurement, which revealed a frequent occurrence of abnormal fluid status (70/89) in patients receiving CRRT, and four types of fluid status corresponded to different clinical conditions and treatment outcomes. Although fluid status was not an independent risk factor for mortality in this study, changes in fluid status and mortality implied potential value of this method for fluid status evaluation in patients receiving CRRT. However, we have to acknowledge the major weakness of the study: the sample size was too small, and the sample size was not calculated, which made the statistics analysis less powerful.

Even though the study included only 89 critically ill patients requiring CRRT and the statistical power of the multivariate analyses of independent risk factors for mortality was weak, the results implied that four classifications of fluid status according to two parameters (BIVA and NT-Pro BNP) correspond to respective clinical condition and treatment outcome. Therefore, the combination of BIVA and NT-pro BNP may have potential value for assessment of fluid status in critically ill patients. However, further studies with large sample sizes are needed to confirm our results.

Previous studies have reported the independent use of BIVA and NT-pro BNP for assessment of fluid or volume status in critically ill patients. This report described that overhydration and abnormal NT-pro BNP levels corresponded to a poorer clinical condition and outcome, which confirmed the roles of these parameters in the assessment of fluid status as reported elsewhere [9-12]. The combination of BIVA and NT-pro BNP was generally used in patients with chronic stable fluid status, such as those receiving maintenance hemodialysis, and was reported to be a good reflection of fluid status with good consistency [13,14]. However, our results showed an inconsistency between the two parameters in 28% of patients receiving CRRT as well as associated differences in clinical condition.

Increasingly, more studies have challenged the role of single use of BNP for assessment of fluid status and prediction of treatment outcome [15-18]. A recent study of 38 patients with severe burns, divided into high and low groups according to BNP level on hospitalization day 3, found that patients with higher BNP levels required less fluid infusion and had lower Sequential Organ Failure Assessment (SOFA) scores and better outcomes. This result was explained by the authors as lower capillary leakage in these patients resulting in retention of intravascular fluid and a consequent increase in BNP level and subsequent better outcome [19]. The prognostic value of BNP in sepsis was investigated in a recent study, which found no differences in BNP concentrations between non-survivors and survivors of septic shock on any study day [20].

The reason for these conflicting results may be due to the reflection of BNP level on only cardiac reaction to current fluid status, which is usually, but not always, consistent with fluid status per se. Therefore, the concept of ‘wet’ and ‘dry’ BNP was proposed [21,22], although there is currently no clear means to differentiate between the two. In the present study, we used the combination of these parameters to categorize fluid status into four types, each corresponding to a different clinical condition and outcome. As a result of the study, type 2 fluid status was associated with much higher BNP levels than type 3 fluid status—591 (203 to 1,949) versus 61 (51 to 65) pmol/L—but conferring a similar mortality with type 3 fluid status. Meanwhile, type 4 fluid status was associated with only a slightly higher BNP level than type 2—817 (294 to 2,549) versus 591 (203 to 1,949) pmol/L—but conferring a mortality much higher than type 2 fluid status. As a summary of the results, among patients with abnormal NT-pro BNP levels, those with and without overhydration (BIVA abnormality) experienced quite different clinical conditions and outcomes. Similarly, for patients with overhydration (BIVA abnormality), there were differences in clinical conditions and outcomes between those with and without abnormal NT-pro BNP levels. Therefore, these results implied a value of the combined use of these two parameters for assessment of fluid status.

The subdivision of fluid status, as presented in this study, may provide a useful aid for the maintenance of fluid balance. Especially, type 3 fluid status (manifested as normal NT-pro BNP and abnormal BIVA) may indicate overhydration but without vascular volume expansion, a condition typically presented by patients with nephrotic syndrome and in some receiving peritoneal dialysis [11]. In regard to critical patients, these parameters may reflect fluid accumulation, but only in extra-vascular spaces, as usually observed in burn and septic shock patients with capillary leakage syndrome caused by a systemic inflammatory response or a fluid shift caused by hypoalbuminemia [19,20]. Under this condition, removal of excess fluid through CRRT should performed only along with infusion of adequate colloids to shift fluid into vascular spaces. Moreover, type 2 fluid status (manifested as normal BIVA and abnormal NT-pro BNP) may imply cardiac dysfunction but no fluid overload. In this situation, proper ultrafiltration by CRRT is essential to alleviate cardiac dysfunction, although over ultrafiltration should be avoided to prevent volume depletion. A recent study investigated the effect of the timing of hemoconcentration on the survival of patients with decompensated heart failure receiving diuretic treatment and reported that those with late versus early hemoconcentration received higher average daily loop diuretic doses (*P* = 0.001), experienced greater weight loss (*P* <0.001), had later transition to oral diuretics (*P* = 0.03), and were discharged earlier (*P* <0.001). Late hemoconcentration conferred a significant survival advantage (*P* = 0.009), whereas early hemoconcentration offered no significant mortality benefit over no hemoconcentration. Although BIVA was not performed in that study, it is expected that patients with late hemoconcentration would be type 4 fluid status, necessitating diuresis and removal of fluid to improve outcome, but that patients with early concentration likely have type 3 fluid status; thus, diuresis and removal of fluid may result in volume depletion but no benefit to outcome. Therefore, water removal via CRRT ultrafiltration should be considerably aggressive only in patients with type 4 fluid status.

Repeated measurement of BIVA and NT-pro BNP in our study helped to elucidate the effect of change in fluid status on treatment outcome. Owing to the small sample size of this study, there is a lack of conclusive results, but some findings were still noteworthy: for patients with type 4 fluid status before CRRT, after intervention, improvement of either BIVA or NT-pro BNP versus no improvement conferred lower mortality; however, those with improvement of both BIVA and NT-pro BNP had the worst outcomes. A possible reason for this poor outcome may be that over ultrafiltration occurred in these patients, resulting in volume depletion. Therefore, the combined use of NT-Pro BNP and BIVA may avoid unnecessary overtreatment of patients with unrecognized normohydration or dehydration resulting in renal impairment.

## Conclusions

Fluid status abnormalities were common among patients receiving CRRT. Different types of fluid status distinguished by BIVA combined with serum NT-pro BNP measurements corresponded to different clinical conditions and treatment outcomes. For patients receiving CRRT, real-time monitoring of fluid status by using BIVA and NT-pro BNP may be useful in fluid management by aiding in the identification of an optimal net ultrafiltration rate during CRRT. However, owing to the small sample size and etiological heterogeneity of the enrolled patients, future studies are needed to confirm the value of the combined use of BIVA and NT-pro BNP.

## Key message

- Different fluid status type prior to start of CRRT in critically ill patients requiring CRRT, as determined by the combined use of BIVA and serum NT-pro BNP measurement, is associated with different clinical conditions and treatment outcomes.

## Abbreviations

AKI: acute kidney injury
APACHE II: Acute Physiology and Chronic Health Evaluation II
BIVA: bioimpedance vector analysis
BNP: B-type natriuretic peptide
CRRT: continuous renal replacement therapy
NT-pro BNP: N-terminal pro-B-type natriuretic peptide
sCr: serum creatinine

## References

[1] Investigators RRTS, Bellomo R, Cass A, Cole L, Finfer S, Gallagher M (2012). An observational study fluid balance and patient outcomes in the Randomized Evaluation of Normal vs. Augmented Level of Replacement Therapy trial. Crit Care Med..

[2] Lombel RM, Kommareddi M, Mottes T, Selewski DT, Han YY, Gipson DS (2012). Implications of different fluid overload definitions in pediatric stem cell transplant patients requiring continuous renal replacement therapy. Intensive Care Med..

[3] Onofriescu M, Hogas S, Voroneanu L, Apetrii M, Nistor I, Kanbay M (2014). Bioimpedance-guided fluid management in maintenance hemodialysis: a pilot randomized controlled trial. Am J Kidney Dis..

[4] Ronco C, Kaushik M, Valle R, Aspromonte N (2012). Peacock WFt: Diagnosis and management of fluid overload in heart failure and cardio-renal syndrome: the ‘5B’ approach. Semin Nephrol..

[5] Arjamaa O (2014). Physiology of natriuretic peptides: The volume overload hypothesis revisited. World J Cardiol..

[6] Valle R, Aspromonte N (2010). Use of brain natriuretic Peptide and bioimpedance to guide therapy in heart failure patients. Contrib Nephrol..

[7] Piccoli A, Rossi B, Pillon L, Bucciante G (1994). A new method for monitoring body fluid variation by bioimpedance analysis: the RXc graph. Kidney Int..

[8] Kidney Disease: Improving Global Outcomes (KDIGO) Acute Kidney Injury Work Group. KDIGO Clinical Practice Guideline for Acute Kidney Injury. Kidney Int Suppl. 2012;2:1–138.

[9] Jeong EG, Nam HS, Lee SM, An WS, Kim SE, Son YK (2013). Role of B-type natriuretic peptide as a marker of mortality in acute kidney injury patients treated with continuous renal replacement therapy. Ren Fail..

[10] Berri RN, Sahai SK, Durand JB, Lin HY, Folloder J, Rozner MA (2012). Serum brain naturietic peptide measurements reflect fluid balance after pancreatectomy. J Am Coll Surg..

[11] Davies SJ, Davenport A (2014). The role of bioimpedance and biomarkers in helping to aid clinical decision-making of volume assessments in dialysis patients. Kidney Int..

[12] Lara TM, Hajjar LA, de Almeida JP, Fukushima JT, Barbas CS, Rodrigues AR (2013). High levels of B-type natriuretic peptide predict weaning failure from mechanical ventilation in adult patients after cardiac surgery. Clinics (Sao Paulo)..

[13] Davenport A (2012). Changes in N-terminal pro-brain natriuretic peptide correlate with fluid volume changes assessed by bioimpedance in peritoneal dialysis patients. Am J Nephrol..

[14] Tapolyai M, Faludi M, Reti V, Lengvarszky Z, Szarvas T, Fulop T (2013). Volume estimation in dialysis patients: the concordance of brain-type natriuretic peptide measurements and bioimpedance values. Hemodial Int..

[15] Agarwal R (2013). B-type natriuretic peptide is not a volume marker among patients on hemodialysis. Nephrol Dial Transplant..

[16] Muller L, Louart G, Teboul JL, Mahamat A, Polge A, Bertinchant JP (2009). Could B-type Natriuretic Peptide (BNP) plasma concentration be useful to predict fluid responsiveness [corrected] in critically ill patients with acute circulatory failure?. Ann Fr Anesth Reanim..

[17] Levitt JE, Vinayak AG, Gehlbach BK, Pohlman A, Van Cleve W, Hall JB (2008). Diagnostic utility of B-type natriuretic peptide in critically ill patients with pulmonary edema: a prospective cohort study. Crit Care..

[18] Maeder MT, Rickenbacher P, Rickli H, Abbuhl H, Gutmann M, Erne P (2013). N-terminal pro brain natriuretic peptide-guided management in patients with heart failure and preserved ejection fraction: findings from the Trial of Intensified versus standard medical therapy in elderly patients with congestive heart failure (TIME-CHF). Eur J Heart Fail..

[19] de Leeuw K, Nieuwenhuis MK, Niemeijer AS, Eshuis H, Beerthuizen GI, Janssen WM (2011). Increased B-type natriuretic peptide and decreased proteinuria might reflect decreased capillary leakage and is associated with a better outcome in patients with severe burns. Crit Care..

[20] Papanikolaou J, Makris D, Mpaka M, Palli E, Zygoulis P, Zakynthinos E (2014). New insights into the mechanisms involved in B-type natriuretic peptide elevation and its prognostic value in septic patients. Crit Care..

[21] Pimenta J, Paulo C, Mascarenhas J, Gomes A, Azevedo A, Rocha-Goncalves F (2010). BNP at discharge in acute heart failure patients: is it all about volemia? A study using impedance cardiography to assess fluid and hemodynamic status. Int J Cardiol..

[22] Parrinello G, Torres D, Paterna S, Di Pasquale P, Licata G (2010). Wet BNP, fluid and hemodynamic status at discharge in acute heart failure. Int J Cardiol..

